# Proteolytic *Lactococcus lactis* and Lipolytic *Enterococcus durans* of Dairy Origin as Meat Functional Starter Cultures

**DOI:** 10.17113/ftb.59.01.21.6872

**Published:** 2021-03

**Authors:** Mirna Mrkonjic Fuka, Ivica Kos, Ana Zgomba Maksimovic, Melita Bacic, Irina Tanuwidjaja

**Affiliations:** 1 Department of Microbiology, University of Zagreb Faculty of Agriculture, Svetošimunska cesta 25, Zagreb, Croatia; 2 Department of Animal Technology, University of Zagreb Faculty of Agriculture, Svetošimunska cesta 25, Zagreb, Croatia

**Keywords:** fermented sausages, native starter cultures, dairy origin, rep-PCR, sensory properties

## Abstract

**Research background:**

As fermentation is an integral feature of both, dry sausage and cheese production, this has led to the evaluation of bacterial cultures *Lactococcus lactis* ssp. *cremoris* (LL8307) and *Enterococcus durans* (ED0207) originally isolated from artisanal Croatian hard type cheese to diversify the range of flavours of dry fermented sausages and to increase their microbiological safety. Both strains were chosen for their high or medium acidifying, proteolytic and/or lipolytic activity and bioprotective potential after step-by-step selection of wild isolates. Therefore, this study aims to evaluate the survival rate of selected starter cultures in wild boar meat sausages during the ripening period of 40 days at a local small-scale facility under artisanal conditions as well as their influence on sausage quality parameters.

**Experimental approach:**

Safety, biotechnological and probiotic properties of twenty-three enterococcal and lactococcal isolates of dairy origin were studied. Based on the results, two best candidates were selected and added to the meat batter during the artisanal wild boar meat sausage preparation where their survival rate, effect on physicochemical, microbiological and sensorial properties and histamine content were evaluated.

**Results and conclusions:**

As revealed by repetitive element-polymerase chain reaction (rep-PCR), native starter cultures survived up to 15 days of ripening and were either absent from (LL8307) or reduced by 80% (ED0207) in final products. The application of native starter cultures rapidly decreased pH (p<0.05), leading to the significantly lower load of *E. coli,* coliforms and *Enterobacteriaceae* in ready-to-eat sausages prepared by the addition of starter cultures (3.04-3.94 log CFU/g) than in the control (3.88-5.00 log CFU/g). Analysis of hedonic test data revealed that some of the sensory traits (odour, flavour, juiciness) of treatments with starter cultures were highly liked by the higher percentage of consumers. The results suggest that these starter cultures would represent a valuable tool to improve the homogeneity of artisanal manufacture and hygienic quality of fermented sausages and can be safely used for food application.

**Novelty and ccientific contribution:**

This is the first study to explore in depth the biotechnological potential of bacterial cultures isolated from artisanal Croatian cheese as functional starter cultures for high-quality game meat sausage production.

## INTRODUCTION

Lactococci and enterococci are usually present in low numbers in artisanal dry fermented sausages and are considered as background microbiota with ambiguous impact on the physicochemical, sensorial and microbiological quality of the sausages (*1*-*3*). Although accepted as minor or even undesirable populations of lactic acid bacteria (LAB) in meat fermentation, particular strains of lactococci and enterococci possess specific and technologically interesting traits that could be used to improve the sausage production, especially sensorial acceptability (*2*, *3*). Of all microbial metabolic pathways, those involved in carbohydrate, lipid and protein conversion contribute largely to the safety, overall acceptability and sensorial properties of the final products. Exogenous proteases and lipases have been successfully used to accelerate the ripening of dry fermented sausages, with the primary aim of reducing production costs (*4*). Likewise, proteolytic and lipolytic strains may significantly influence the ripening process and the quality of the ready-to-eat sausages. However, the final result is difficult to predict as, under certain conditions, excess proteolysis and lipolysis may result in bitter and metallic off-flavour due to the presence of bitter peptides or excess oxidation of lipids (*5*).

Spontaneously fermented sausages prepared from wild boar (*Sus scrofa*) hold an important place in the game meat production and consumption. They are generally characterized by distinctive sensory properties, differing from those of ’domestic’ meat and are mostly darker, stronger tasting and often tougher, reflecting the fact that these animals are raised in the wild and feed on naturally growing plants (*6*, *7*). Spontaneously fermented sausages are mostly manufactured by local producers following traditional procedures, without nitrate or starter culture addition. Consequently, they are recognized and appreciated as authentic traditional products, and their production is anticipated to increase as consumers demand more organic food of particular taste and flavour.

Although dry fermented sausages are considered microbiologically stable, in the case of elevated numbers of pathogens in raw material or inadequate production conditions, their safety can be compromised (*8*, *9*). Several zoonotic agents and enteric bacteria can be transmitted from wildlife to humans by contaminated game meat, particularly if the intestine is ruptured by shot pellets or during evisceration. The risk especially increases under poorly controlled environmental conditions or if small game meat producers do not follow good manufacturing practice. However, to provide high-quality products and possibly expand the production of traditional meat sausages, high food safety standards have to be met. The possibilities are being investigated intensively, and frequently involve the use of starter cultures with proven antimicrobial and technological properties that might ensure both, microbiological safety and unimpaired sensorial quality of nitrate-free sausages (*10*-*12*). Besides, in terms of functional food, additional properties of starters such as probiotic potential are much appreciated (*13*). However, if potential starter culture does not multiply inside the meat product or/and is outcompeted by wild microbiota, its metabolic activity in sausages is minimized, despite *in vitro* potential and high performance under laboratory conditions (*14*).

As fermentation is an integral feature of both, dry sausage and cheese production, this has led to the evaluation of bacterial cultures *Lactococcus lactis* ssp. *cremoris* (LL8307) and *Enterococcus durans* (ED0207) originally isolated from artisanal Croatian hard type cheese to diversify the flavour range of dry fermented sausages and to increase their microbiological safety. Both strains were chosen for their high or medium acidifying, proteolytic and/or lipolytic activity and bioprotective potential after step-by-step selection of wild isolates. Therefore, this study aims to evaluate the survival rate of selected starter cultures in wild boar meat sausages during the ripening period of 40 days at a local small-scale facility under artisanal conditions. Moreover, their influence on the microbiological, physicochemical and sensory properties as well as histamine content of sausages was determined.

## MATERIALS AND METHODS

### Origin and strain selection

All tested bacterial cultures were isolated from spontaneously fermented traditional Croatian cheese in a previous study (*15*) and deposited in the culture collection of the Department of Microbiology, Faculty of Agriculture, University of Zagreb. All Gram-positive, coagulase-negative cocci prevously identified as *Enterococcus durans* and *Lactococcus lactis* (*N*=23) were subjected to a step-by-step selection including a detailed safety, technological and antimicrobial analyses.

### Safety, technological, antimicrobial and probiotic potential

Firstly, the strain (*N*=23) safety traits including the haemolysis, susceptibility to antibiotics as well as histamine and virulence determinants were determined. The haemolytic capacity of the strains was analyzed on Columbia blood agar (bioMérieux, Crappone, France) where *Bacillus cereus* DSM 6791 served as a positive control. The susceptibility to clinically relevant antibiotics including ampicillin (2 and 10 µg), clindamycin (2 µg), gentamicin (10 µg), tetracycline (5 and 10 µg), erythromycin (2 and 15 µg), vancomycin (5 µg) and chloramphenicol (30 µg) was measured by the standardized agar disc diffusion method using BBL™ Sensi-Disc™ antimicrobial susceptibility test discs (Becton, Dickinson and Company, Rungis, France) as described elsewhere (*16*). To detect the genes encoding for the production of histamine (*hdc*), a PCR assay was performed by following the protocol of De Las Rivas *et al.* (*17*). Enterococci were additionally screened for the presence of virulence factors including aggregation substance (*agg*), gelatinase (*gelE*), cytolysin (*cylM, cylB*), cytolysin activator (*cylA*), enterococcal surface protein (*esp*), and sex pheromones (*cpd* and *cob*) as reported before (*18*).

The acidifying activity of selected strains was determined by measuring the pH in brain heart infusion (BHI) broth (Biolife, Milan, Italy) inoculated with an appropriate strain in duplicates. The measurements were taken at the beginning (0 h) and after 24 h of incubation at 37 °C for enterococci and 30 °C for lactococci. The pH was measured by combined pH electrodes (InPro^®^ 3030; Mettler Toledo, Greifensee, Switzerland) that were disinfected after each use with 3% HCl. The acidification rate was calculated according to Jamaly *et al.* (*19*) and expressed as ΔpH.

Lipolytic activity was screened on tributyrin agar (Oxoid, Hampshire, UK) by disc diffusion method. Bacterial suspensions (10 μL) at a cell concentration corresponding to the 0.5 McFarland standard were inoculated on sterile cellulose discs (Bio-Rad Laboratories, Hercules, CA, USA) previously placed on the agar. Plates were then incubated for 3 days at 37 °C for enterococci and 30 °C for lactococci. Proteolytic activity was tested in the same way as lipolytic activity, except instead of tributyrin agar, BHI agar supplemented with skimmed milk (1.5%) was used (*16*). The diameter of clear zone was measured and expressed as a mean value in mm and compared to the lypolitic and proteolytic activity of *Pseudomonas fluorescens* WCS 417r.

The peptidolytic activity was analyzed in the presence of chromogenic peptide *N*-succinyl-Ala-Ala-Pro-Phe-*p*-nitroanilide (Sigma-Aldrich, Merck, St Louis, MO, USA). The release of *p*-nitroanilide (*p*NA) by the action of bacterial peptidases was measured at 410 nm, as described by de Giori and Hébert (*20*). Results were expressed as μM *p*NA.

Antimicrobial activity was tested against seven indicator bacteria, *Salmonella enterica* (DSM 14221), *Listeria innocua* (ATCC 33090), *Escherichia coli* (ATCC 25922), *Staphylococcus aureus* ssp. *aureus* (DSM 20231), *Brochotrix thermospachta* (LMG 17208), *Weissella viridescens* (DSM 20410) and *Bacillus cereus* (DSM 6791) using a modified agar streak-spot technique (*21*). The difference between the colony diameter of indicator bacteria and the diameter of control colony was estimated. The results were expressed as follows: no inhibition (<1 mm), weak inhibition (1–2 mm), pronounced inhibition (2–4 mm), very strong inhibition (>4 mm) and complete inhibition (no growth). The antimicrobial activity of strains was additionally tested using the cell-free supernatant neutralized with 1 M NaOH and filtered through a 0.22-µm membrane filter (Merck, Darmstadt, Germany) and the agar well diffusion method as described previously (*22*).

The probiotic potential of the best starter culture candidates *Enterococcus durans* (ED0207) and *Lactococcus lactis* ssp. *cremoris* (LL8307) with respect to safety, technological and antimicrobial traits was determined. In order to estimate the survival rate of strains in simulated gastric and intestinal conditions, the protocol of Doleyres *et al.* (*23*) with slight modifications as described by Mrkonjic Fuka *et al.* (*16*) was followed. Briefly, to simulate the gastric digestion, 0.5% NaCl and 0.3% pepsin solution (Sigma Chemicals, Merck, St Louis, MO, USA) was used and the pH was adjusted to pH=2.5 with 1 M HCl. For cell survival under simulated intestinal conditions, 0.4% bile salt and 0.2% pancreatin solution (Sigma Chemicals, Merck) was applied. The survival of each strain was evaluated after the incubation under the above mentioned conditions and subsequent plating on BHI agar for colony forming units (CFU) count. Aggregation was evaluated as described by Del Re *et al*. (*24*) using 4 mL of cell suspension corresponding to viable counts of approx. 10^8^ CFU/mL. The cell suspension was vortexed for 10 s and the absorbance was measured on UV/VIS spectrophotometer (PerkinElmer, Waltham, MA, USA) at 610 nm after 5 h of incubation at room temperature.

In addition, the growth of potential starter cultures (ED0207 and LL8307) at temperatures relevant for artisan (12 °C) and industrial (25 °C) sausage production and in the presence of typical stressors such as 3.0 and 6.0% NaCl at pH=4.5 was determined by visually assessing the turbidity after 48 h of incubation.

### Biomass preparation

Before the application of potential starter cultures ED0207 and LL8307, they were grown aerobically in BHI broth (100 mL) at 30 °C for 24 h. Each strain was harvested by centrifugation at 8000×*g* for 5 min. Cell pellets were then resuspended in 100 mL of sterile skimmed milk solution (1.5%) and were added to the meat batter. Total viable count (TVC) of inoculum was estimated on plate count agar (PCA) (Merck) after 24 h of incubation at 30 °C.

### Formulation and sampling of sausages

In this study, three batches of fermented sausages were prepared each with 25 kg of meat batter. All batches were made from a mixture of domestic pig (*Sus scrofa domesticus* L.) meat (50%) and wild boar (*Sus scrofa* L.) meat (50%) and the following ingredients were added: salt (1.9%), red chilli peppers (0.5%), garlic (0.3%), red sweet peppers (0.2%), sugar (0.2%), and black pepper (0.1%). Spices were added dried and grounded. Selected native starter cultures were applied resuspended in sterile skimmed milk solution: *E. durans* ED0207 in treatment A, *Lactococcus lactis* ssp. *cremoris* LL8307 in treatment B, and treatment C was used as a non-inoculated control (no starter cultures were added). Meat batter was filled in natural casings (pig’s small intestine) with a 38 mm diameter, and the sausages were allowed to ripen in a drying chamber, with four smoking treatments under traditional conditions for 40 days. Sausages were randomly distributed in the fermentation/ripening chamber. Temperature and relative humidity were monitored every 30 min using data-logger LOG 32 TH (Dostmann Electronic GMBH, Wertheim-Reicholzheim, Germany). Three sausages were sampled per one sampling time and analyzed independently at 0, 4, 7, 15 and 40 days for the evaluation of the survival rate, pH, water activity and microbiological quality, and the final products were tested for histamine and sensory properties.

### Water activity and pH

Water activity (*a*_w_) was determined using a portable analyzer, HygroPalm HP23-AW-A equipped with an HC2-AW probe (Rotronic AG, Bassersdorf, Switzerland) and pH values were measured using a portable pH-meter IQ 150 (IQ Scientific Instruments, San Diego, CA, USA) equipped with a spear type glass electrode BlueLine 21pH (Schott AG, Mainz, Germany).

### Microbiological analysis of spontaneously fermented sausages

Twenty-five grams of sample (without casing) of each sausage were aseptically transferred to sterile plastic pouches and homogenized in a sterile saline solution (0.85%) using a Stomacher Lab-Blender 400 (Seward Medical, London, UK). Appropriate dilutions of the sample homogenates were prepared in duplicates in peptone water and inoculated in growth media for the enumeration and detection of particular microbial groups. *Enterobacteriaceae* were determined on Violet Red Bile Glucose agar (VRBG; Merck) after incubation at 37 °C for 24 h according to ISO 21528-2:2004 (*25*). *Staphylococcus aureus* was determined on Baird Parker agar (Labo-Life Sàrl, Pully, Switzerland) supplemented with egg yolk tellurite emulsion (20%; VWR International AG, Dieticon, Switzerland) and incubated at 37 °C for 48 h. Chromocult® Coliform agar ES (Merck) was used for the differentiation and enumeration of *E. coli* and coliforms at 37 °C for 24 h, according to ISO 4832:2006 (*26*). Yeasts and moulds were enumerated on Dichloran Rose-Bengal Chloramphenicol agar (DRBC; Biolife, Milan, Italy) supplemented with chloramphenicol at 25 °C for 5 days under aerobic conditions according to ISO 21527-2:2008 (*27*). *Salmonella* spp. and *Listeria monocytogenes* were detected according to ISO 6579:2002/AMD 1:2007 (*28*) and ISO 11290-1:1996/AMD 1:2004 (*29*), respectively. The presence of enterococci was determined on kanamycin esculin azide agar (KAA; Biolife) after incubation at 37 °C for 48 h. *Lactobacillus* spp. were isolated on MRS (de Man, Rogosa, and Sharpe, Merck) agar supplemented with vancomycin and bromocresol green (LAMVAB) (*30*) and *Lactococcus* spp. on M17 agar (Merck) under anaerobic conditions after 72 h at 30 °C. Approximately 15 colonies from the KAA and M17 media were randomly selected and puriﬁed from each spontaneously fermented sausage batch on sampling day (0, 7, 15 and 40 days; *N*=150) and were subjected to DNA extraction.

### Extraction of DNA and identification of strains

The template DNA was extracted from the KAA and M17 isolates following the protocol of Wizard Genomic DNA Purification Kit (Promega, Madison, WI, USA). The concentration and purity of the DNA were determined by a NanoDrop ND-1000 spectrophotometer (Thermo Fisher Scientific Inc., Waltham, MA, USA).

For the identification at the species level, part of the 16S rRNA gene was amplified and sequenced (Macrogen, Seoul, South Korea) using the universal bacterial primer sets 27f and 1401R (*31*, *32*). The obtained sequences were analyzed with the Nucleotide Basic Local Alignment Search tool (BLASTn) (*33*) and a minimum sequence identity of 98% was chosen as a criterion for species identification.

In addition, all collected KAA and M17 isolates (*N*=150) were identified at the strain level and genotyped to evaluate their survival efficiency during the ripening of sausages. The molecular fingerprinting and strain-specific identification were performed by the repetitive rep–PCR using (GTG)_5_ oligonucleotide as a primer (*34*) as described elsewhere (*21*). The rep-PCR patterns were analyzed and compared to those obtained from the applied starter cultures *Lactococcus lactis* ssp. *cremoris* (LL8307) and *Enterococcus durans* (ED0207) Only 100% identical fingerprinting patterns were considered as the same strain.

### Histamine content

An enzyme-linked immunosorbent assay (ELISA) kit for histamine determination (RIDASCREEN®) was provided by R-Biopharm (Darmstadt, Germany). All reagents were included in the commercial kit and the assay was performed following the manufacturer’s instructions.

### Sensory properties

Sensory analysis was performed by a hedonic test using 112 consumers among students and faculty staff and the basic socio-demographic characteristics were as follows: 54% female and 46% male; 65% under age 35, 28% between 35 and 55 and 7% above age 55; 6% with low income, 70% with medium income and 24% with high income; 48% lived in rural and 52% in urban areas. Samples were scored on a 10-point structured scale where 0 meant ’extremely disliked’ and 9 meant ’extremely liked. Six sensory traits were evaluated: cross-section, odour, flavour, hardness, juiciness and overall likeability. Samples were cut by a knife to 2 mm thickness under 90° angle, and the presentation order was defined as a completely balanced block design. Subjects were placed in separate booths and were instructed to use tap water and unsalted bread as palate cleansers before every sample.

### Statistical analysis

The data were subjected to analysis of variance (one-way ANOVA) by SAS® Studio University Edition v. 3.8 (*35*) and Tukey’s test with p≤0.05 was used for the evaluation of statistically significant differences. For the analysis of microbial count, histamine content, pH and water activity value, a general linear model with treatment as the fixed effect was used, while for the analysis of sensory data, the assessor was added as a random effect in a MIXED model. Results are presented as mean value±standard error.

## RESULTS AND DISCUSSION

### Characterization of starter cultures

In selecting strains with optimal properties, safety was the first selection criterion followed in our study. Although all testing isolates showed no haemolytic activity, most of them did not pass safety characterization (82%) as they were resistant to one or more antibiotics (mostly tetracycline, clindamycin and rifampicin) or possessed more than one virulence determinants (data not shown). The remaining four candidates that passed the safety characterization were tested for acidifying, proteolytic and lipolytic activities as the most important technological properties of starter cultures in fermented meat products because of their influence on texture and flavour development (*36*). Of tested strains (*N*=4), one was low acidifier (ΔpH˂1.5) and was discarded from further analysis, and three showed medium (ΔpH=1.5-2.0) or high (ΔpH>2.0) acidification potential (*37*). The lipolysis was noticed only for one of the three tested isolates. All three candidates showed some degree of proteolytic or peptidolytic activity; however, only one candidate stood out with high values considering proteolytic and peptidolytic potential. Based on the above results, two candidate strains *Lactococcus lactis* ssp. *cremoris* (LL8307) and *Enterococcus durans* (ED0207) were selected and further analyzed (Table 1). These strains exhibited a medium (ED0207; ΔpH=1.50) to high acidification potential (LL8307; ΔpH=2.32), pronounced peptidolytic activity measured as concentration of *p*-nitroanilide (*p*NA) released from the chromogenic peptide (S-Ala) (21 578±39 μM for LL8307), or noticeable lipolytic activity analyzed on agar with tributyrin (ED0207).

**Table 1:** Overview of technological properties, antagonistic activity and growth in different ecophysiological conditions of native starter culture

Strain	Acidification ΔpH after 24 h	Lypolitic activity^1^	Proteolytic activity		Antimicrobial activity^3^	*t*=12 °C, time=48 h^4^				*t*=25 °C, time=48 h^4^				Probiotic potential/%		Auto-aggregation after 5 h/%
Casein^2^	Chromogenic *c*(pNA)/μM	*w*(NaCl)/%		pH=4.5	Control	*w*/NaCl)/%		pH=4.5	Control	Gastric	Duodenum
		3	6			3	6				
*Enterococcus durans*																
ED0207	1.51±0.03	+ +	+	8492±707	Sa/L/Ec/St/W	+ +	+	+	+ + +	+ + +	+ + +	+ + +	+ + +	65.3±0.5	81.7±3.2	5.9±0.5
*Lactococcus lactis*																
LL8307	2.38±0.01	-	+++	21578±39	Sa/St/W	-	-	+ +	+ + +	+ + +	-	+ +	+ + +	56.9±2.4	16.4±1.2	27.0±1.4

Data were expressed as mean value±standard error (SE). ^1^The diametar of clear zone was measured and expressed as: no lypolitic activity (6.0-7.0 mm), +=weak lypolitic activity (7.1-8.0 mm), ++=pronounced lypolitic activity (8.1-9.0 mm), and +++=very strong lypolitic activity (>9.0 mm). ^2^The diametar of clear zone was measured and expressed as: no proteolytic activity (6.0-8.0 mm), +=weak proteolytic activity (8.1-10.0 mm), ++=pronounced proteolytic activity (10.1-12.0 mm), and +++=very strong proteolytic activity (>12.0 mm). ^3^Antimicrobial activity was tested against: Sa=*Salmonella enterica* ssp. *enterica* (DSM 14221), L=*Listeria innocua* (ATCC 33090), Ec-*Escherichia coli* (ATCC 25922), St=*Staphylococcus aureus* ssp. *aureus* (DSM 20231), Bt=*Brochotrix termospachta* (LMG 17208), W=*Weissella viridescens* (DSM 20410), Bc=*Bacillus cereus* (DSM 6791). All postive results detected were classifed as weak inhibition. ^4^Growth in different ecophysiological conditions was determined by visually assesing the turbidity: -=no growth, +=weak growth, ++=pronounced growth, and +++=very strong growth

Both strains grew at temperatures usually used for traditional or industrial meat fermentation (12 and 25 °C) and exhibited physiological traits characteristic for each bacterial group. Strain LL8307 did not grow at 6% NaCl irrespective of the incubation temperature, and much reduced growth of both strains was noticed at 12 °C, even resulting in the absence of growth at pH=4.5 (ED0207) or in 3 or 6% NaCl (LL8307).

The weak antimicrobial capacity (˂1 cm diameter) of both strains was observed against Gram-positive and -negative bacteria. A somewhat broader spectrum of antimicrobial activity of strain ED0207 was noticed (against *Salmonella* spp., *S. aureus*, *E. coli*, *Listeria innocua* and *Weisella viridescens*) than of LL8307 (against *Salmonella* spp., *S. aureus* and *Weisella viridescens*). However, this activity was not detected using the cell-free supernatants, suggesting that the inhibitory effect was most probably a result of the production of antimicrobially active metabolites such as organic acids or hydrogen peroxide, rather than the ability to produce bacteriocins (*14*).

However, mechanisms underlying the activity of LAB strains against bacterial pathogens appear to be multifactorial (*38*) and they are well known in the GI tract. As such, LAB can prevent the adhesion of pathogens by competing for the binding sites on the intestinal epithelial cells and consequently, reduce the colonization, thereby preventing the onset of infection (*39*, *40*). However, in order to extend beneficial effects in the intestine, besides a high initial level of viable microorganisms, a probiotic needs to achieve adequate biomass through growth and aggregation. Consequently, the ability to aggregate is a desirable property of probiotics (*41*).

Both tested strains in our study showed a similar surviving rate under gastric conditions, 65.31 and 56.89% for ED0207 and LL8307 respectively. However, remarkable differences (p˂0.01) were noticed under simulated duodenal conditions, where 81.70% of the added ED0207 and only 16.38% of LL8307 survived. However, despite the huge growth reduction of LL8307 in the presence of bile salt and pancreatin, more than 10^8^ cells (from the initial 2.9·10^9^) still survived the passage through the duodenum, which should be adequate biomass to exhibit probiotic efficiency (*42*). However, the autoaggregation ability of both strains was either low (25% for LL8307) or absent (5% for ED0207), meaning low or no capacity of adhesiveness and persistence in the GI tract (*24*).

### Biomass production and the survival rate of the inoculated starter cultures

To ensure that a starter culture is effective, appropriate bacterial biomass should be produced and applied to the meat batter. The level of culture to be added depends on the product specifications, but a high viable number of cells is generally used, ranging from 5 to 9 log CFU/g (*14*). In this study, the viable cell counts of the inoculum added to the meat batches A and B were comparable: (9.32±0.06) log CFU/mL of ED0207 (batch A) and (9.38±0.08) log CFU/mL of LL8307 (batch B), corresponding to the 6.98 and 6.92 log CFU/g of meat respectively.

**Fig. 1:**
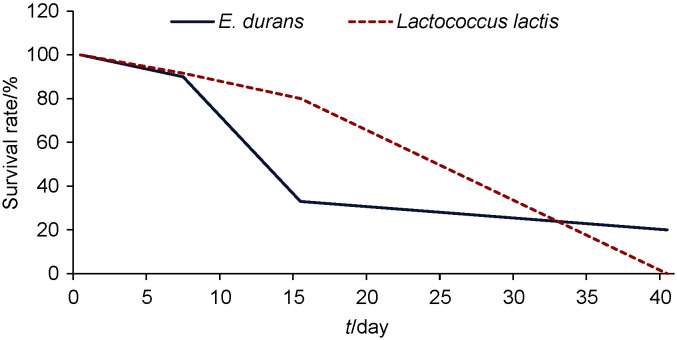
Survival rate of applied starter cultures *Enterococcus durans* ED0207 and *Lactococcus lactis* ssp. *cremoris* LL8307

However, the ability of the starter culture to compete with the natural microbiota and to undertake the metabolic activities to improve the nutritional and microbiological quality of fermented sausages is based on its ability to multiply inside the meat product, not only on high biomass applied (*14*). Therefore, to follow the succession of a particular strain in a complex microbial community, rep-PCR fingerprinting of KAA and M17 isolates on days 0, 7, 15 and 40 was performed. Based on the rep-PCR analysis, a different pattern for the survival rate of the applied starters was noticed (Fig. 1). On day seven, a 10% reduction of survival rate of both strains was observed. However, a remarkable difference was noticed on day 15 when less than 33% of isolates were assigned to ED0207 and 80% to LL8307. On day 40, only 20% of enterococcal isolates were identified as ED0207 and none of the LL8307 was detected. At the same time, the number of both strains and on both days was higher than 5.2 log CFU/g, which led to the conclusion that wild enterococci and lactococci outcompeted the added starter cultures during the prolonged ripening period. Moreover, in many similar studies, the survival rate of the applied starter culture was followed by counting microbes on selective agar media typical for a particular microbial group (*43*, *44*). Because of the findings in our study, such an approach is not satisfactory and should be always conducted with a relevant fingerprinting method that allowed tracking of the particular strain of interest.

### Microbial evolution, physicochemical properties and histamine content

**Fig. 2:**
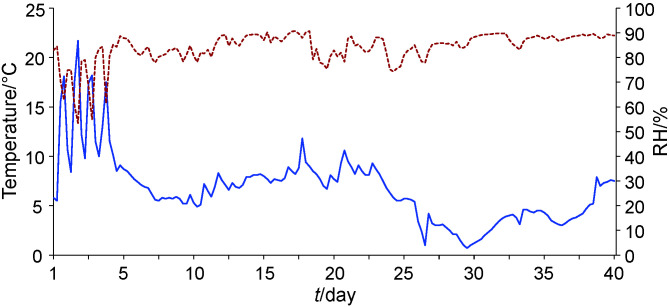
Temperature (blue line) and relative humidity (RH, red line) during sausage production

The whole process of sausage production was closely monitored but only partly controlled as it is usually performed in artisanal production. Three distinctive temperatures and relative humidity values were characterized (Fig. 2). During the first five days, the temperature varied from 5.5 to 22 °C (with four peaks during smoking) and relative humidity was between 54 and 87% (lower during the smoking phase). In the next 21 days, the temperature was between 5 and 12 °C and relative humidity between 74 and 90%. In the third period, the temperature was affected by the extreme outer conditions, with lower values close to 1 °C and the maximum was 8 °C. The third period was also characterized by very stable but high relative humidity (79–90%), not usually present in artisanal production. These data show that artisanal production, which is affected by outer weather conditions, is variable, as previously found by Zgomba Maksimovic *et al.* (*8*), and this highlights the need for standardization in other aspects like raw material and ingredient quality and/or starter cultures application.

The mass of sausages was on average 544.50 g, and the production ended when mass loss reached about 30%. Water activity (*a*_w_) and pH were measured at different time of the production (Table 2) and the change of *a*_w_ and pH values was typical for fermented artisanal wild boar sausages (*8*, *45*). At the beginning of production, the *a*_w_ was between 0.97 and 0.98 in all three treatments, which slightly declined after seven days (0.93-0.95) and reached values between 0.86 and 0.87 in the final products (Table 2). The signiﬁcant differences in *a*_w_ between treatments with starter cultures and control samples were found only on days 7 and 15 of production (p>0.05). A similar trend was detected for pH values, which were uniform at the beginning and the end of production. Significant differences between control and experimental treatments were established on day 7, when the effect of starter culture addition was seen as lower pH values. These results correspond to the *a*_w_ values that were significantly lower in control treatment on day 7 than in experimental treatments (0.93 *vs* 0.95), meaning that microbial growth is slightly reduced in the control treatment, resulting in the reduced formation of organic acids.

**Table 2:** Microbiological analysis on selective media, water activity (*a*_w_) and pH values of sausages produced by the application of native starter cultures

Treatment	*t*/day	*N*/(log CFU/g)								*a*_w_	pH
LAMVAB (lactobacilli)	M17 (lactococci)	KAA (enterococci)	VRBG (*Enterobacteriaceae*)	DRBC (yeasts)	CCA (coliforms)		CCA (*E.coli*)
A	0	3.80.1	(5.34±0.02)^b^	(6.4±0.1)^a^	4.68±0.03	(4.04±0.08)^a^	(4.74±0.03)^b^		3.81±0.03	0.97±0.00	5.7±0.0
	7	8.2±0.1	(6.17±0.02)^b^	(7.51±0.01)^a^	(3.82±0.01)^c^	5.01±0.02	(4.05±0.04)^b^		(3.08±0.06)^c^	(0.95±0.00)^b^	(4.97±0.01)^b^
	15	8.24±0.07	(6.1±0.1)^a^	(6.59±0.05)^a^	(3.84±0.01)^a^	6.13±0.37	(4.68±0.02)^ab^		(3.32±0.05)^c^	(0.93±0.00)^b^	5.00±0.01
	40	(7.40±0.06)^ab^	(4.26±0.06)^b^	(5.3±0.4)^a^	(3.44±0.02)^c^	(6.0±0.1)^b^	(3.94±0.03)^a^		(3.04±0.03)^b^	0.86±0.01	5.25±0.01
B	0	3.6±0.1	(6.24±0.06)^a^	(4.37±0.01)^b^	4.60±0.05	(3.69±0.08)^b^	(5.33±0.05)^a^		4.12±0.08	0.98±0.00	5.73±0.01
	7	8.28±0.01	(7.31±0.04)^a^	(5.30±0.06)^b^	(5.31±0.02)^a^	4.94±0.02	(5.68±0.05)^a^		(3.69±0.05)^b^	(0.95±0.00)^b^	(5.03±0.01)^b^
	15	8.20±0.05	(6.3±0.2)^a^	(5.37±0.05)^b^	(5.00±0.02)^a^	6.26±0.07	(4.54±0.08)^b^		(3.58±0.04)^b^	(0.92±0.00)^ab^	5.07±0.01
	40	(7.32±0.07)^b^	(5.20±0.05)^a^	(3.78±0.01)^b^	(3.84±0.03)^b^	(6.04±0.05)^b^	(3.64±0.04)^b^		(3.15±0.04)^b^	0.86±0.01	5.16±0.02
C	0	3.69±0.05	(3.1±0.1)^c^	(3.40±0.01)^c^	4.54±0.02	(3.31±0.06)^c^	(5.37±0.01)^a^		4.1±0.1	0.98±0.00	5.74±0.03
	7	8.1±0.1	(4.04±0.08)^c^	(4.84±0.02)^c^	(4.84±0.01)^b^	5.01±0.08	(4.06±0.06)^b^		(4.01±0.08)^a^	(0.93±0.00)^a^	(5.15±0.02)^a^
	15	8.19±0.05	(4.94±0.03)^b^	(4.17±0.06)^c^	(5.07±0.08)^a^	6.91±0.03	(4.89±0.02)^a^		(3.89±0.06)^a^	(0.91±0.00)^a^	5.10±0.01
	40	(7.69±0.07)^a^	(3.42±0.01)^c^	(3.77±0.00)^b^	(5.00±0.03)^a^	(6.33±0.05)^c^	(4.90±0.02)^c^		(3.88±0.01)^a^	0.87±0.01	5.27±0.02

A=*Enterococcus durans* ED0207, B=*Lactococcus lactis* ssp. *cremoris* LL8307, C=non-inoculated control. LAMVAB=MRS (de Man, Rogosa and Sharpe) agar supplemented with vancomycin and bromocresol green (*30*). KAA=kanamycin aesculin azide agar, VRBG=violet red bile glucose agar, CCA=chromogenic coliforms agar. Data were expressed as mean value±SE. Values with different letters in superscript within sampling day are significantly different between treatments (p<0.05)

The number of lactobacilli isolated on the LAMVAB medium on day zero was comparable in all three treatments (3.69-3.75 log CFU/g). A similar trend was noticed during the fermentation and ripening indicating that applied starter cultures did not affect the growth of wild lactobacilli in our trial. The number of lactobacilli reached the level above 8 log CFU/g in all treatments and remained there until the end of ripening, when it decreased to 7.32–7.69 log CFU/g (p<0.05) (Table 2).

Although *Salmonella* spp., *Staphylococcus aureus* and *Listeria monocytogenes* were not detected, the elevated number of coliforms, *E. coli* and *Enterobacteriaceae* is of special concern. The initial high number of respective microbes (above 4 log CFU/g of *Enterobacteriaceae* or *E. coli* and above 5 log CFU/g of coliforms) was only partially overcome by the application of starter cultures. Although a significantly lower number (p<0.05) of coliforms, *E. coli* and *Enterobacteriaceae* was noticed in ready-to-eat sausages produced with starter cultures than in the control, the amount of respective microbial groups still exceeded the limits set by the Health Protection Agency (HPA) (*46*) in all samples, except in treatment B (Table 2). The findings above suggest poor raw meat hygiene, most probably due to the faecal contamination at shooting or evisceration. As such, the power of starter cultures to decrease the number of undesirable microbiota to acceptable values is much reduced when an elevated number of potential pathogens is present in the raw material.

The content of biogenic amines in sausages can be influenced by a combination of many factors, such as ripening conditions, formulation, pH, temperature, additives, diameter and salt/water ratio, as well as the proteolytic and decarboxylase activities of developed microbiota (*47*). Although there is no legislation dealing with the content of histamine in dry fermented sausages, the mass fraction between 100 and 200 mg/kg seems to be acceptable for many countries (*48*). However, the histamine mass fraction measured in our study was far below these values and comparable in all three treatments: 3.31, 4.27 and 4.54 mg/kg in treatment A, C and B, respectively.

### Sensorial quality

**Fig. 3:**
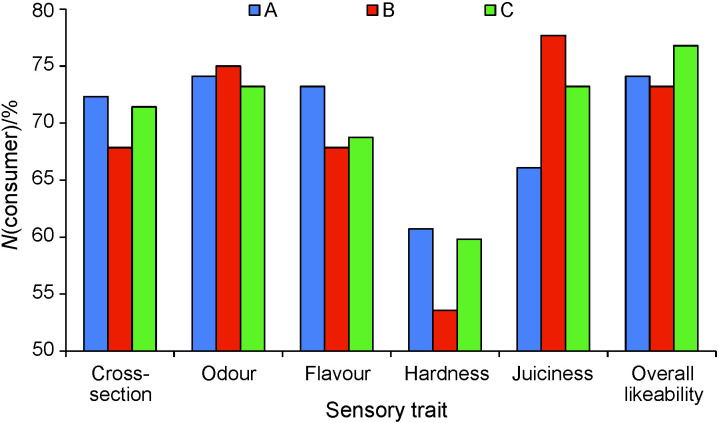
Percentage of consumers scoring sensory traits with high likeability. A=*Enterococcus durans* ED0207, B=*Lactococcus lactis* ssp. *cremoris* LL8307, C=non-inoculated control

Analysis of hedonic test data revealed that sensory traits of sausages did not differ significantly between treatments, as shown in Table 3. This is similar to the research conducted by Talon *et al.* (*49*) who found that the addition of autochthonous starters did not affect the overall aroma and flavour of the sausages, but there were some effects on texture traits. Contrary, the preference ranking test (*45*) revealed that the addition of bacterial starter cultures had a significant and positive effect on the sensory score. Within this research, all sensory traits had an average value around 7, except hardness, which was rated with lower values. Because average values were similar, frequency analysis was performed and scores equal to or higher than 7 were counted (Fig. 3). This was described as the percentage of consumers scoring sensory traits with high likeability. It was established that all traits were highly liked by more than 50% consumers with hardness being the least liked, and overall likeability being the most liked trait. There were some differences between treatments and traits. Some of the traits (odour, flavour, juiciness) of experimental treatments A and B were highly liked by the higher share of consumers, suggesting the positive contribution of starter cultures to sensory traits. This can lead to the conclusion that the addition of starter cultures did not have any adverse effect on sensory traits and can even improve likeability in some groups of consumers. It is well known that the use of well-selected strains that generate high amounts of aroma components could improve the sensory quality and/or accelerate the meat fermentation process, as stated by Leroy *et al.* (*50*). The application of these high-yielding starters was not performed in this investigation, and a significant effect on sensory traits was not established. This can be further elaborated with the addition of a large amount of intensive spices (garlic and red hot pepper) into meat batter, which can dominate the aroma profile. Finally, the sensory hedonic testing was performed on a large number of untrained consumers (*N*=112) who usually increase variability and are unwilling to give maximum scores like trained panellists.

**Table 3:** Sensory traits of sausages produced by the addition of native culture starters

Trait	Treatment		
A	B	C
Cross-section	7.0±0.2	7.0±0.1	7.1±0.1
Odour	7.2±0.2	7.3±0.1	7.1±0.1
Flavour	7.2±0.1	6.9±0.2	7.1±0.2
Hardness	6.71±0.21	6.3±0.2	6.6±0.2
Juiciness	7.07±0.14	7.4±0.1	7.2±0.1
Overall likeability	7.2±0.1	7.2±0.1	7.2±0.1

A=*Enterococcus durans* ED0207, B=*Lactococcus lactis* ssp. *cremoris* LL8307, C=non-inoculated control

Data were expressed as mean value±SE

## CONCLUSIONS

In this study safety, biotechnological and probiotic properties of twenty-three enterococcal and lactococcal isolates of dairy origin were analyzed. Based on the obtained results, two best candidates showing high or medium acidifying, proteolytic and/or lipolytic activity as well as bioprotective and probiotic potential were selected and applied into the meat batter and evaluated for their survival rate and effect on physicochemical, microbiological and sensorial properties as well as histamine content of artisanal wild boar meat sausages.

The present study demonstrates that strains of lactococci and enterococci of dairy origin might be able to inhibit potential pathogens and improve sensory properties, and at the same time maintain the final pH within the range for non-acid/low-acid fermented sausages. However, the poor hygienic quality of meat can only partially be overcome by the application of starter cultures. The high hygienic quality of meat must be therefore the standard goal for high-quality sausage production.

Both applied native starter cultures survived in the sausages until day 15 of production; however, were either absent from or present in a very low number in final products, which leads to the conclusion that wild enterococci and lactococci outcompete our starter strains and, consequently, their probiotic features can be excluded. Finally, this points to the need for tracking of specific strains in starter formulation by fingerprinting method through the whole process of production, as counting of a particular microbial group on selective agar media is not sufficient.
